# CAPTEM in Metastatic Well-Differentiated Intermediate to High Grade Neuroendocrine Tumors: A Single Centre Experience

**DOI:** 10.1155/2019/9032753

**Published:** 2019-02-20

**Authors:** Arvind Sahu, Michael Jefford, Julia Lai-Kwon, Alesha Thai, Rodney J. Hicks, Michael Michael

**Affiliations:** ^1^Department of Cancer Medicine, Peter MacCallum Cancer Centre, 305 Grattan Street, Melbourne, VIC 3000, Australia; ^2^Department Of Medical Oncology, Goulburn Valley Health, Shepparton VIC 3630, Australia; ^3^Department of Rural Health, Melbourne Medical School, The University of Melbourne, Shepparton, VIC, Australia; ^4^Neuroendocrine Unit, Peter MacCallum Cancer Centre, 305 Grattan St, Melbourne VIC 3000, Australia; ^5^Department of Cancer Imaging, Peter MacCallum Cancer Centre, 305 Grattan St, Melbourne VIC 3000, Australia

## Abstract

**Introduction:**

Capecitabine-temozolomide (CAPTEM) has significant activity in patients (pts) with metastatic low grade pancreatic neuroendocrine tumors (NETs). However, there is limited data regarding its activity in pts with metastatic well-differentiated intermediate and high grade pancreatic and nonpancreatic NETs. The objective of this study was to assess the functional imaging response, survival, and tolerability of CAPTEM in this population.

**Methods:**

A retrospective audit of pts with metastatic well-differentiated intermediate (WHO grade 2) or high grade (WHO grade 3) NETs treated at Peter MacCallum Cancer Centre between March 2013 and March 2017. Pts received capecitabine 750 mg/m^2^ orally twice daily (bd) from days1 to 14 and temozolomide 100 mg/m^2^ bd from days 10 to 14 every 28 days. Data regarding functional imaging response, progression-free and overall survival, and toxicities was collected.

**Results:**

Thirty-two pts received a median of 6 cycles (range: 2-16) of CAPTEM for grade 2 (n=21, 66%) or grade 3 (n=11, 34%), Ki67 <55% (n= 7, 21.9%) or Ki67 ≥55% (n= 4, 12.5 %) NET. Primary site included gastroenteropancreatic (n= 17, 53%), lung (n= 12, 37.5%), and unknown origin (n = 3, 9.4%). Twenty-two percent received CAPTEM as first-line therapy. After a median of 31 months of follow-up, the two-year overall survival (OS) was 42%, with a median OS of 24 months. There was a trend towards improved median progression-free survival (PFS) in pts with low grade 3 (Ki67<55%) versus high grade 3 (Ki67 ≥55%) NETs (15 vs 4 months, p= 0.11). Ten (31.3%) experienced grade 3/4 toxicity, with nausea (15.6%), thrombocytopaenia (12.5%), and fatigue (9.4%) the most common toxicities reported.

**Conclusion:**

CAPTEM has significant activity in patients with metastatic grades 2 and 3 NETs with manageable toxicity. The PFS benefit observed in the grade 3 subgroup with Ki67<55% warrants further evaluation in a larger randomized trial.

## 1. Introduction

Neuroendocrine tumors (NETs) are a rare, heterogeneous group of tumors classified according to their site of origin, histology, Ki-67 index, and mitotic count [1].

Metastatic well-differentiated low and intermediate grade NETs are typically managed with long-acting somatostatin analogues (SSAs) or biological agents (everolimus and sunitinib) [2, 3]. In contrast, patients with grade 3 disease are typically managed with chemotherapy. Small cell lung cancer regimens (such as platinum and etoposide) are typically used, but response rates tend to be significantly lower ranging from 30-50% in patients with Ki67 >55% to 15% where Ki 67 is 20-55% [4–8]. The NORDIC study in particular identified a subgroup characterized by a Ki-67 <55% that had a very low objective response rate to C/E chemotherapy but, nevertheless, a better prognosis than those with a Ki-67 >55%. This and other data support the concept of a group of well-differentiated G3 NET they may have more in common with G1-2 NET than with G3 NEC [9, 10].

The role of cytotoxic chemotherapy for patients with well-differentiated intermediate to high grade NETs has evolved over time, though the ideal regimen remains uncertain. Consensus guidelines from the European Neuroendocrine Tumor Society (ENETS) and North American Neuroendocrine Society (NANETS) do not include specific recommendations for a particular chemotherapy regimen. Historically, streptozocin (STZ) regimens (STZ ± doxorubicin or 5-fluorouracil) were the only approved regimens for patients with metastatic pancreatic NETs (pNETs) [11–13]. Response rates in retrospective studies ranged from 6 to16% with substantial grade 3 and 4 toxicities [14, 15]. More recent studies have examined other chemotherapy regimens, potentially with less toxicity. Retrospective studies have demonstrated efficacy for temozolomide (with or without capecitabine) [16–22], oxaliplatin (FOLFOX) [23], and irinotecan (FOLFIRI) [24] with response rates ranging between 20 and 35%.

Capecitabine and temozolomide (CAPTEM) has demonstrated significant activity in patients with metastatic well-differentiated pancreatic NETs [25, 26]. However, its utility in patients with well-differentiated intermediate to high grade pancreatic or nonpancreatic NETs is not well established. Being a completely oral regimen, better patient convenience is one of the biggest ostensible advantages of this regimen.

Peter MacCallum Cancer Centre is the largest comprehensive cancer centre in Australia, a state and national referral centre for NETs patients as well as an ENETS Centre of Excellence. We present our experience with the CAPTEM regimen in patients with metastatic intermediate (grade 2, Ki 67 3-20%) and high grade (grade 3, Ki67 > 20%) NETs, including responses based upon functional imaging, survival, and toxicity.

## 2. Methods

### 2.1. Study Design/Patients

This study was a retrospective audit of all patients with metastatic intermediate (WHO grade 2) or high (WHO grade 3) grade NETs who were treated with CAPTEM between March 2013 and March 2017. All patients were deemed ineligible for peptide receptor radionuclide therapy (PRRT) due to the presence of disease sites that lacked sufficient somatostatin receptor expression. Fluorine-18 fluorodeoxyglucose positron emission tomography combined with computed tomography (FDG PET/CT) was routinely performed in the pretreatment evaluation of all patients in addition to SSTR imaging, generally using gallium-68 DOTA-octreotate PET/CT. FDG-avidity or objective evidence of progression of unresectable disease on functional or anatomic imaging was considered adequate indications for active treatment according to treatment pathway guidelines established within the NET Service.

### 2.2. Data Collection

Demographic, clinical, and radiological data was collected from patients' electronic medical records. Demographic data included the patient's age, gender, and Eastern Cooperative Oncology Group (ECOG) performance status. Clinical data included date of diagnosis, site of primary NET, Ki67 index, tumor grade, site of metastases, prior therapies received, and date of progression and/or death. Data regarding CAPTEM administration (date of initiation and cessation, number of cycles received, and toxicities as per CTCAE criteria [27]) was also collected.

### 2.3. Treatment

All patients received capecitabine 750 mg/m^2^ bd orally on days 1–14 and temozolomide 100 mg/m^2^ bd orally on days 10–14 on a 28 day cycle as per standard schedule with premdications and antiemetics [28]. All patients received dexamethasone 8mg and 5HT_3_ antagonist on day 10-day 16. Treatment was commenced with neutrophil count > 1.5 and platelet count > 100 and recovery from prior toxicity to grade 1. Dose reductions were done by 20% if febrile neutropenia or significant grade 4 or grade 3 nonhematologic toxicity as per standard protocol [18, 28].

### 2.4. Assessments

The primary endpoints of the study were progression-free survival (PFS) (defined as the time from the date of initiation of CAPTEM to the date of first evidence of progression on FDG PET/CT, which was used as the primary therapeutic response assessment modality) and overall survival (OS) (defined as the time from the date of initiation of CAPTEM to the date of death due to any cause).

The secondary endpoints were response rates on functional imaging, toxicity and the association between extent of prior therapy and functional response. Responses on functional imaging were according to PET response criteria in solid tumors (PERCIST 1.0) [29].

### 2.5. Statistical Analysis

All data was analyzed using SPSS software version 25. Descriptive statistics including median, frequency, and percentage for categorical variables were used to describe age, gender distribution, site of primary, treatment, and response to treatment. Survival analysis was performed using Kaplan-Meier estimates and log-rank test for bivariate comparisons. The collected data for the purpose of the statistical analysis to support the findings of this study are available from the corresponding author upon request.

## 3. Results

Thirty-two patients were eligible [male: n=13, female: n=19, and median age at enrolment: 58 (range: 31-76)]. Baseline characteristics are shown in Table 1. Grade 3 tumors were divided on the basis of Ki67 into low grade 3 (Ki67< 55%) and high grade 3 (Ki67 ≥ 55%). Previous SSA therapy was not considered a line of therapy. The median time from diagnosis until onset of treatment with CAPTEM was 17 months (range: 1-132 months). Median number of cycles received was 6 (range 2-16). MGMT methylation status was not available as it was not routinely evaluated in our patients with NET.

### 3.1. Functional Imaging Response

The best functional imaging response achieved was complete metabolic response in four patients (12.5%) while 11 (34.4%) achieved a partial metabolic response and five (15.6%) had stable disease. The overall objective response rate was 46.9% and the objective disease control rate was 62.5%.

### 3.2. Survival Outcomes

**Figure 1:**
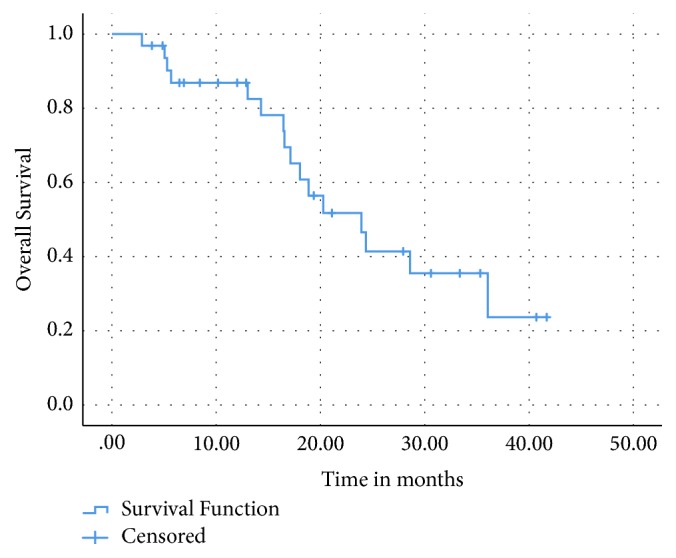
Overall survival for entire cohort.

**Figure 2:**
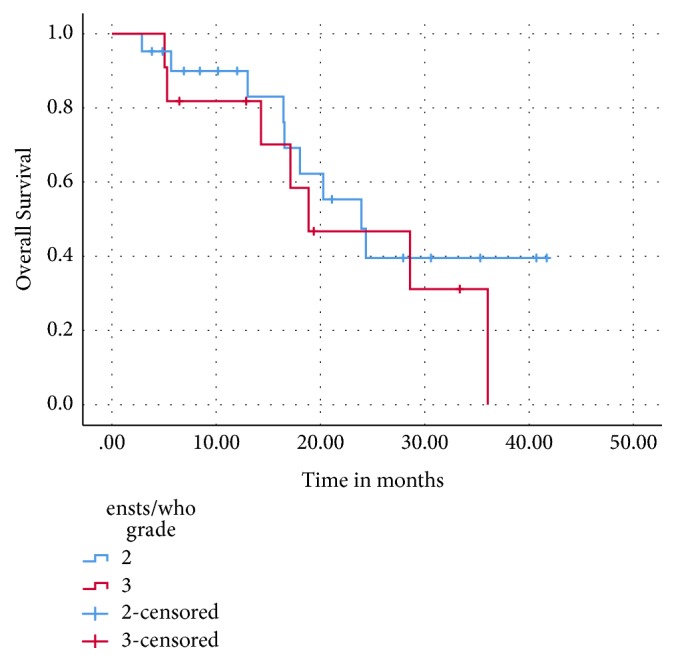
Overall survival: grade 2 versus grade 3 metastatic NETs.

After a median of 31 months of follow-up, the median OS for the entire cohort was 24 months (95% CI: 17.1- 30.8) and the two-year OS was 42% (Figure 1). The median OS for patients with grade 2 NETs was 24 months versus 19 months for grade 3 NETs (p = 0.42) (Figure 2).

**Figure 3:**
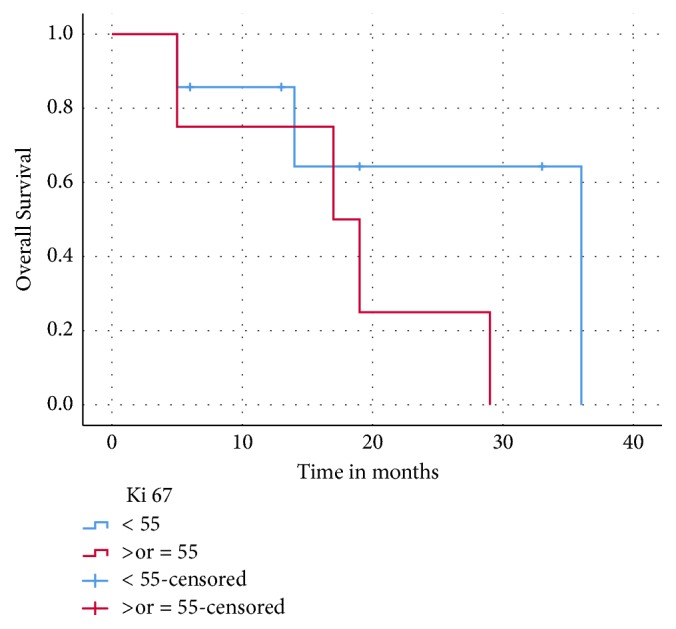
Overall survival: grade 3 NETs (Ki67 <55% versus ≥55%).

Patients with low grade 3 (Ki67 20-55%) had a higher OS compared to those with high grade 3 NETs (Ki67 ≥ 55%) (36 versus 17 months, p = 0.169) (Figure 3), as did patients who received CAPTEM as first-line therapy compared to those who had received prior lines of systemic therapy (29 versus 20 months, p= 0.49). As shown, these differences were not statistically significant. There was no difference in OS with respect to the site of primary (gastroenteropancreatic versus lung) or the site of metastasis.

**Figure 4:**
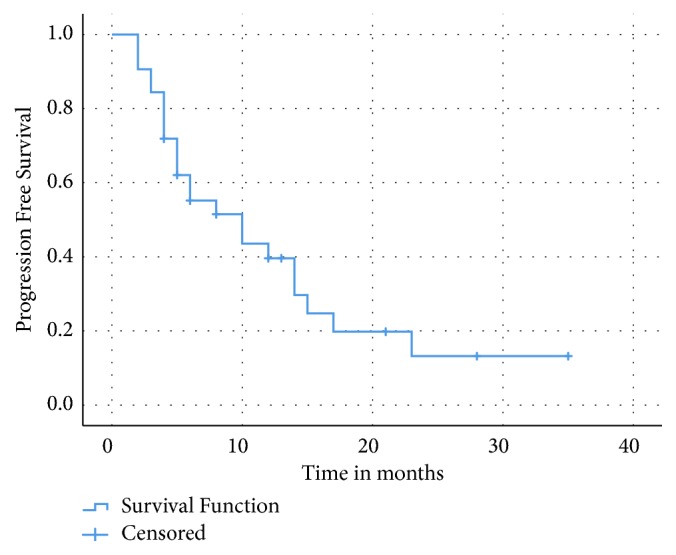
Progression-free survival: entire cohort.

**Figure 5:**
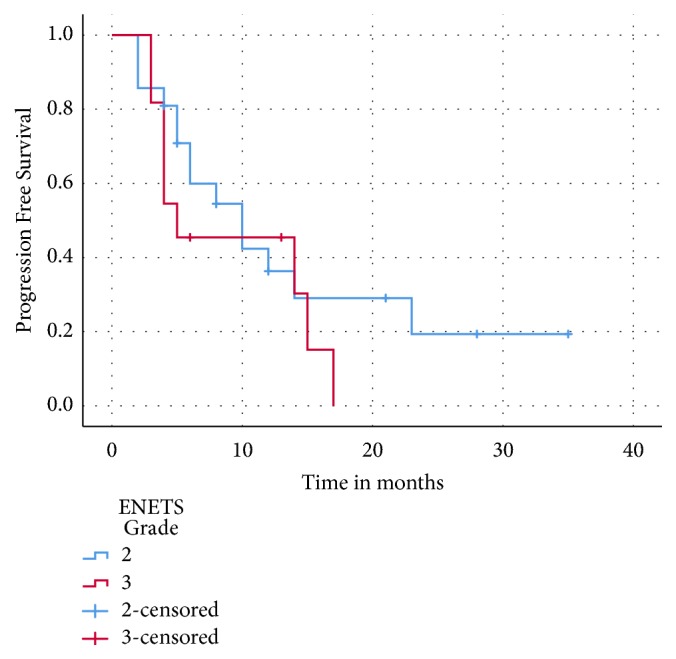
Progression-free survival: grade 2 versus grade 3 metastatic NETs.

The median PFS for the entire cohort was 10 months (95% CI: 3.7- 16.2) (Figure 4). The median duration of response was 21 months (95% CI: 8.8- 33.1). There was a trend towards an improved median PFS for grade 2 versus grade 3 NET but this was not statistically significant (10 versus 5 months, p= 0.3) (Figure 5). There was also a trend towards improved median PFS in patients with low grade 3 (15 versus 4 months, p = 0.117) and for patients who received CAPTEM as first-line therapy compared to those who had received prior lines of therapy (17 versus 8 months, p= 0.3).

### 3.3. Toxicity

The toxicity profile of CAPTEM is shown in Table 2. Grade 3 and 4 toxicities were seen in 10 patients (31.3%). The most commonly reported grade 3 and 4 toxicities were nausea (15.6%), thrombocytopenia (12.5 %), fatigue (9.4%), anaemia (9.4%), febrile neutropenia (9.4%), diarrhoea (6.3%), vomiting (3.1%), and mucositis (3.1%). Treatment discontinuation due to toxicity occurred in 4 patients (12.5%) (3 for febrile neutropenia and 1 for grade 4 multiple nonhematologic toxicities) while 16 (50%) of patients continued the regimen till progression. None of the patients were continued on a single drug as maintenance.

## 4. Discussion

The optimal treatment for patients with metastatic intermediate and high grade NETs is not established. A few retrospective and prospective studies have demonstrated activity of CAPTEM in patients with advanced pancreatic neuroendocrine tumors (PNETs) and non-PNETs. There has been considerable difference among the reports with regards to the line of therapy, site of primary (pNET or non-PNET), response evaluation (conventional imaging vs functional imaging), and even dosing schedule of CAPTEM regimen. Although the numbers are small, this study highlights the improved survival with CAPTEM in first line which needs to be further evaluated in a prospective manner. We observed an objective response rate of 46.9% and a disease control rate of 62.5%. The median PFS was 10 months with a 2-year OS of 42%. In an exploratory analysis of patients who received CAPTEM in the first line, we demonstrated a median PFS of 17 months.

This is consistent with previously reported data. In a series of 30 patients with metastatic well- or moderately-differentiated tumors pancreatic neuroendocrine tumors who received CAPTEM in the first-line setting, the response rate was 70% with a median PFS of 18 months [16]. Similarly, in another retrospective study of patients with metastatic intermediate and high grade NETs (pancreatic and nonpancreatic included) receiving CAPTEM, median PFS was 15.9 months with CAPTEM when used in first line as compared to 3.1 months when used in subsequent lines of therapy [30].

There is a wide variation in the clinical course and outcomes of patients with Ki-67 values greater than 20%. This is consistent with the results of our study which suggests that patients with Ki 67 20-55% had improved survival outcomes as compared to patients with Ki 67> 55%.

A single prospective phase II study has examined CAPTEM in patients with a variety of metastatic NETs [28]. An interim analysis of 28 out of a planned 38 patients with typical and atypical carcinoids, pituitary, and pancreatic NETs demonstrated an overall response rate of 43% with stable disease in 54%. The median PFS was greater than 20 months and OS greater than 25.3 months. Similarly, the E2211 study in metastatic low and intermediate grade pancreatic NETs demonstrated an improvement in OS. It also reported the longest PFS for any pancreatic NET-directed therapy [31]. This study randomized 144 pretreated patients with metastatic low and intermediate grade pancreatic NETs comparing temozolomide (200 mg/m2 PO QD days 1-5) vs. temozolomide plus capecitabine (T 200 mg/m2 PO QD days 10-14; C 750 mg/m2 PO BID days 1-14. The median PFS for CAPTEM in this study was 22.7 months whereas the median OS was not reached.

CAPTEM appears to be relatively well tolerated, with nausea, thrombocytopenia, and anaemia the most common grade 3 or 4 toxicities. Being a retrospective study, the toxicities might not have been adequately captured so it may not be representative for the chemotherapy regimen. However out results are consistent with previous retrospective reports of CAPTEM toxicity [16, 18, 25]. The median number of cycles delivered in our review was six. The median number of cycles of CAPTEM in earlier studies ranged between 6 and 10 [16, 26, 31].

The audit has its limitations of being retrospective and of a small sample size as expected of uncommon tumors. Conversely, the selection of patients for treatment on the basis of functional imaging and, specifically, the requirement for sites of disease with FDG-avidity that lack SSTR expression is likely to have biased the population of G3 NEN towards a poorer prognostic group given the adverse outcomes in such patients [32, 33]. It is also difficult to have accurate radiological response data with RECIST evaluation in retrospective studies. However in our study functional imaging has supported the survival parameters and has confirmed that the results of our study are to be in line with other retrospective studies and add to the growing body of evidence for the use of CAPTEM in this rare malignancy.

## 5. Conclusion

CAPTEM is an oral chemotherapy regimen which is associated with objectives responses and promising survival outcomes in metastatic well-differentiated intermediate and high grade NETs, including in non-pNET tumors. In particular, survival outcomes in the first-line setting and the low grade 3 (Ki67<55%) subgroup are promising and require further investigation. The toxicity profile of CAPTEM is favorable compared to streptozocin-based regimens and other targeted agents and comparable to other chemotherapy regimens. The ENETs guidelines currently recommend this regimen for patients with intermediate and high grade NETs as a second line therapy [34]. Randomized, prospective trials are needed to further evaluate this regimen, particularly in the subgroups identified, and establish a standard of care in this rare malignancy.

## Figures and Tables

**Table 1:** Baseline characteristics.

Baseline characteristics		Number (%)
		n=32
Median age (years)		58
Range		31-76

Gender	Male	13 (40.6)
	Female	19 (59.4)

ECOG performance status	1	30 (93.8)
	2	2 (6.2)

Primary site	Gastroenteropancreatic	17 (53.1)
	(i) Pancreatic	14 (43.8)
	(ii) Non Pancreatic	3 (9.3)
	Lung	12 (37.5)
	Unknown	3 (9.4)

ENETS Tumor Grade	2 (Ki 67 – 2-20)	21 (65.6)
	3 (Ki 67> 20)	11 (34.4)
	Ki67 >20- <55	7 (21.9)
	Ki67 > 55	4 (12.5)

Sites of metastasis	Liver	23 (72)
	Bones	20 (62)
	Lymph nodes	20 (62)
	Lung	13 (41)
	Soft tissue deposits	9 (28)
	Brain	4 (12)
	Breast	2 (6)
	Spleen	1 (3)
	Peritoneal	1 (3)
	Adrenal	1 (3)

Prior Treatment	No Treatment	7 (21.9)
	Number of prior regimens	
	One line of prior therapy	13 (40.6)
	2 or more lines of prior therapy	12 (37.5)
	SSA	16 (50)
	Streptozocin/5-FU	3 (9.4)
	Carboplatin and etoposide	10 (31.3)
	FOLFIRI	1 (3)
	Sunitinib	1 (3)
	Everolimus	5 (15.6)
	PRRT	7 (21.9)

Abbreviations

ECOG - Eastern Cooperative Oncology Group

ENETS - European Neuroendocrine Tumor Society

SSA - somatostatin analog

5-FU – 5 – Fluorouracil

PRRT - Peptide receptor radionuclide therapy

**Table 2:** Toxicity profile.

Toxicity	Grade 3/4	Any grade
	N (%)	N (%)
Nausea	5 (15.6)	30 (93)

Thrombocytopenia	4 (12.5)	10 (31)

Anemia	3 (9.4)	11 (34)

Febrile neutropenia	3 (9.4)	-

Fatigue	3 (9.4)	32 (100)

Diarrhoea	2 (6.3)	12 (37.5)

Vomiting	1 (3.1)	18 (56)

Mucositis	1 (3.1)	11 (34)

Hand foot syndrome	0	14 (43.8)

## Data Availability

The demographic and clinical data collected for the purpose of the statistical analysis to support the findings of this study are available from the corresponding author upon request.
